# Taxa: An R package implementing data standards and methods for taxonomic data

**DOI:** 10.12688/f1000research.14013.2

**Published:** 2018-09-11

**Authors:** Zachary S.L. Foster, Scott Chamberlain, Niklaus J. Grünwald

**Affiliations:** 1 Department of Botany and Plant Pathology, Oregon State University, Corvallis, OR, 97331, USA; 2 rOpenSci, University of California, Berkeley, CA, 94720, USA; 3 Horticultural Crops Research Laboratory, USDA Agricultural Research Service, Corvallis, OR, 97330, USA

**Keywords:** R language, taxonomy, taxa, R package, rOpenSci, metacoder, taxize

## Abstract

The taxa R package provides a set of tools for defining and manipulating taxonomic data. The recent and widespread application of DNA sequencing to community composition studies is making large data sets with taxonomic information commonplace. However, compared to typical tabular data, this information is encoded in many different ways and the hierarchical nature of taxonomic classifications makes it difficult to work with. There are many R packages that use taxonomic data to varying degrees but there is currently no cross-package standard for how this information is encoded and manipulated. We developed the R package taxa to provide a robust and flexible solution to storing and manipulating taxonomic data in R and any application-specific information associated with it. Taxa provides parsers that can read common sources of taxonomic information (taxon IDs, sequence IDs, taxon names, and classifications) from nearly any format while preserving associated data. Once parsed, the taxonomic data and any associated data can be manipulated using a cohesive set of functions modeled after the popular R package dplyr. These functions take into account the hierarchical nature of taxa and can modify the taxonomy or associated data in such a way that both are kept in sync. Taxa is currently being used by the metacoder and taxize packages, which provide broadly useful functionality that we hope will speed adoption by users and developers.

## Introduction

The R statistical computing language is rapidly becoming the leading tool for scientific data analysis in academic research programs ( Tippmann, 2015). One of the reasons for R’s popularity is how easy it is to develop and install extensions called R packages, relative to other programming languages. There are now more than 10,000 packages on the Comprehensive R Archive Network (CRAN), over 1,300 packages on Bioconductor ( Gentleman *et al.*, 2004), and countless more on GitHub.

The recent increases in the affordability and effectiveness of high-throughput sequencing has led to a large number of ecological datasets of unprecedented size and complexity. The R community has responded with the creation of numerous packages for ecological data analysis and visualization, such as vegan ( Oksanen *et al.*, 2013), phyloseq ( McMurdie & Holmes, 2013), taxize ( Chamberlain & Szöcs, 2013), and metacoder ( Foster *et al.*, 2017). Taxonomic information is often associated with these large data sets and each package encodes this information differently. Some store taxonomic classification as a table with ranks as columns (e.g. phyloseq), some store it as simple character vectors (i.e. plain text) or column/row names, leaving it up to the user to decide on the details on how taxa in the classification are distinguished (e.g. vegan), and some store it as a list of tables with one classification in each table (e.g. taxize). Since each package tends to have a unique focus, it is common to use multiple packages on the same data set but converting between formats can be difficult. Considering how recently these large taxonomic data sets have become commonplace, it is likely that many more packages that use taxonomic information will be created.

Without a common data standard, using multiple packages with the same data set requires constant reformatting, which complicates analyses and increases the chance of errors. Package maintainers often add functions to convert between the formats of other popular packages, but this practice will become unsustainable as the number of packages dealing with taxonomic data increases. Even if a conversion function exists, doing the conversion can significantly increase the time needed to analyze very large data sets, like those generated by high-throughput sequencing. In addition, not all formats accommodate the same types of information, so conversion can force a loss of information.

The sources of taxonomic data, typically online databases, also vary in how they are encoded. Reference sequence databases used in ecology research often have taxon names in the headers separated by some character, but the details differ. For example, the popular Greengenes database ( McDonald *et al.*, 2012) for prokaryotic 16S sequences encodes classifications as follows:



                    k__Bacteria; p__Cyanobacteria; c__Synechococcophycideae...
                


In contrast, the SILVA database ( Yilmaz *et al.*, 2014) uses:



                    Bacteria;Proteobacteria;Gammaproteobacteria...
                


And the Ribosomal Database Project (RDP) ( Cole *et al.*, 2014) has the ranks and taxon names intermixed with the same separator:



                    Root;rootrank;Fungi;domain;Ascomycota...
                


These minor differences, while not a problem for humans to understand, mean that different code must be used to read each type. Also, this information is often intermixed with other information in the same header, like the sequence ID or description of the organism further complicating parsing. In other cases, a classification might not be supplied at all, but just a taxon name (e.g. *Homo sapiens*), sequence ID, or taxon ID, as is done in sequences downloaded from GenBank:



                    >AC005336.1 Homo sapien chromosome 19
                


In this case the classification must be looked up using tools like the taxize package, but to do that the relevant information must be extracted from the rest of the header.

Taxa is a new R package that defines classes and functions for storing and manipulating taxonomic data. It is meant to provide a solid foundation on which to build an ecosystem of packages that will be able to interact seamlessly with minimal hassle for developers and users. It also provides highly flexible functions to read in data (i.e. parsers) from diverse formats, allowing it to be used with the ever-changing and proliferating selection of file formats used by biologists. The classes in taxa are designed to be as flexible as possible so they can be used in all cases involving taxonomic information. Complexity ranges from low level classes used to store the names of taxa, ranks, and databases to high-level classes that can store multiple data sets associated with a taxonomy. In particular, the taxmap class is designed to hold any type of arbitrary, user-defined data associated with taxonomic information, making its applications limitless. In addition to the classes, there are associated functions for manipulating data based on the dplyr philosophy ( Wickham & Francois, 2015). These functions provide an intuitive way of filtering and manipulating both taxonomic and user-defined data simultaneously. In combination with flexible parsers and classes, this allows for taxa to be used to subset complicated data/files based on their associated taxonomic information.

## Methods

### Implementation

**Figure 1.**
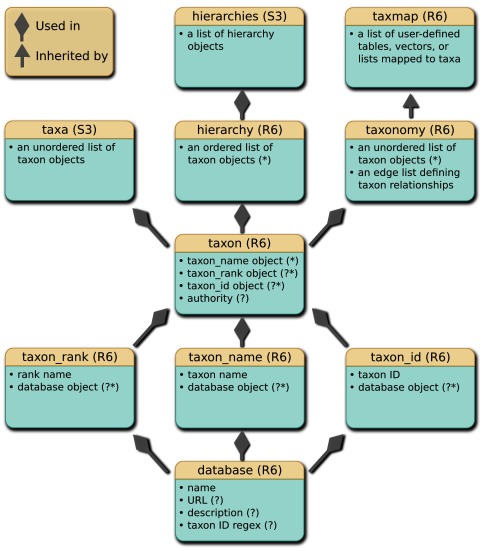
A class diagram representing the relationship between classes implemented in the taxa package. Diamond-tipped arrows indicate that objects of a lower class are used in a higher class. For example, a database object can be stored in the taxon_rank, taxon_name, or taxon_id objects. A standard arrow indicates that the lower class is inherited by the higher class. For example, the taxmap class inherits the taxonomy class. An asterisk indicates that an object (e.g. a database object) can be replaced by a simple character vector. A question mark indicates that the information is optional.

***The basic classes*.** Taxa defines some basic taxonomic classes and functions to manipulate them ( Figure 1). The goal is to use these as low-level building blocks that other R packages can use. The database class stores the name of a database and any associated information, such as a description, its URL, and a regular expression matching the format of valid taxon identifiers (IDs):



                        taxon_database(
  name = "ncbi",
  url = "
                            http://www.ncbi.nlm.nih.gov/taxonomy",
  description = "NCBI Taxonomy Database",
  id_regex = "*")
#> <database> ncbi
#>   url: 
                            http://www.ncbi.nlm.nih.gov/taxonomy
#>   description: NCBI Taxonomy Database
#>   id regex: *
                    


The classes taxon_name, taxon_id, and taxon_rank store the names, IDs, and ranks of taxa and can include a database object indicating their source:



                        taxon_name("Poa", database = "ncbi")
#> <TaxonName> Poa
#>   database: ncbi
taxon_rank(name = "species", database = "ncbi")
#> <TaxonRank> species
#>   database: ncbi
taxon_id(12345, database = "ncbi")
#> <TaxonId> 12345
#>   database: ncbi
                    


 All of the classes mentioned so far can be replaced with character vectors in the higher-level classes that use them. This is convenient for users who do not have or need database information. However, using these classes allows for greater flexibility and rigor as the taxa develops; new kinds of information can be added to these classes without affecting backwards compatibility and the database objects stored in the taxon_name, taxon_id, and taxon_rank classes can be used to verify the integrity of data, even if data from multiple databases are combined. These classes are used to create the taxon class, which is the main building block of the package. It stores the name, ID, and rank of a taxon using the taxon_name, taxon_id, and taxon_rank classes. The taxa class is simply a list of taxon objects with a custom print method (i.e. the function controlling how it is displayed when printed to the console).

***The hierarchy and taxonomy classes.*** The taxon class is used in the hierarchy and taxonomy classes, which store multiple taxa ( Figure 1). The hierarchy class stores a taxonomic classification composed of nested taxa of different ranks (e.g. *Animalia*, *Chordata*, *Mammalia*, *Primates*, *Hominidae*, *Homo*, *sapiens*). Each taxon is stored as a taxon object in a list in the order they appear in the classification, from most inclusive to most specific. The hierarchies class is simply a list of hierarchy objects with a custom print method. The hierarchies class has the convenience of each hierarchy being independent, making it easy to subset by index or name, but it could also waste memory by storing multiple copies of the more coarse taxa (e.g. *Animalia*) that are likely to appear in many hierarchy objects. The taxonomy class is a more memory-efficient alternative that can store the same information.

The taxonomy class stores multiple taxa in a tree structure representing a taxonomy. The individual taxa are stored as a list of taxon objects and the tree structure is stored as an edge list representing subtaxa-supertaxa relationships. The edge list is a two-column table of taxon IDs that are automatically generated for each taxon. Using automatically generated taxon IDs, as opposed to taxon names, allows for multiple taxa with identical names. For example, *Achlya* is the name of an oomycete genus as well as a moth genus. It is also preferable to using taxon IDs from particular databases, since users might combine data from multiple databases and the same ID might correspond to different taxa in different databases. For example, “180092” is the ID for *Homo sapiens* in the Integrated Taxonomic Information System, but is the ID for *Acianthera teres* (an orchid) in the NCBI taxonomy database. The tree structure of the taxonomy class uses less memory than the same information saved as a table of ranks by taxa, since the information for each taxon occurs in only one instance. It also does not require explicit rank information (e.g. “genus” or “family”).

***The taxmap class.*** The taxmap class inherits the taxonomy class and is used to store any number of data sets associated with taxa in a taxonomy ( Figure 1). A list called “data” stores any number of lists, tables, or vectors that are mapped to all or a subset of the taxa at any rank in the taxonomy. Therefore, the raw data used to make the object (and any other data associated with it) can be included in the taxmap object itself in its original form. In the case of tables, the presence of a “taxon_id” column containing unique taxon IDs indicates which rows correspond to which taxa. Lists and vectors can be named by taxon IDs to indicate which taxa their elements correspond to. When a taxmap object is subset or otherwise manipulated, these IDs allow for the taxonomy and associated data to remain in sync. The taxmap also contains a list called “funcs” that stores functions that return information based on the content of the taxmap object. In most functions that operate on taxmap objects, the results of built-in functions (e.g. n_obs), user-defined functions, and the user-defined content of lists, vectors, or columns of tables can be referenced as if they are variables on their own, using non-standard evaluation (NSE). NSE is a technique used to make functions more convenient to use by interpreting things like variable names in a function call differently than they would be outside the function call or in other functions not using NSE. Any value returned by the all_names function can be used in this way. This greatly reduces the amount of typing needed and makes the code easier to read.

***Manipulation functions.*** The hierarchy, hierarchies, and taxa classes have a relatively simple structure that is easily manipulated using standard indexing (i.e. using [, [[, or $), but the taxonomy and taxmap classes are hierarchical, making them much harder to modify. To make manipulating these classes easier, we have developed a set of functions based on the dplyr data manipulation philosophy. The dplyr framework provides a consistent, intuitive, and chain-able set of commands that is easy for new users to understand. For example, filter_taxa and filter_obs are analogs of the dplyr filter function used to subset tables.

One aspect that makes dplyr convenient is the use of NSE to allow users to refer to column names as if they are variables on their own. The taxa package builds on this idea. Since taxmap objects can store any number of user-defined tables, vectors, lists, and functions, the values accessible by NSE are more diverse. All columns from any table and the contents of lists/vectors are available. There are also built-in and user-defined functions whose results are available via NSE. Referring to the name of the function as if it were an independent variable will run the function and return its results. This is useful for data that is dependent on the characteristics of other data and allows for convenient use of the magrittr %>% piping operator. For example, the built-in n_subtaxa function returns the number of subtaxa for each taxon. If this was run once and the result was stored in a static column, it would have to be updated each time taxa are filtered. If there are multiple filtering steps piped together using %>%, a static “n_subtaxa” column would have to be recalculated after each filtering to keep it up to date. Using a function that is automatically called when needed eliminates this hassle. The user still has the option of using a static column if it is preferable to avoid redundant calculations with large data sets.

Unlike dplyr’s filter function, filter_taxa works on a hierarchical structure and, optionally, on associated data simultaneously. By default, the hierarchical nature of the data is not considered; taxa that meet some criterion are preserved regardless of their place in the hierarchy. When the subtaxa option is TRUE, all of the subtaxa of taxa that pass the filter are also preserved and when supertaxa is TRUE, all of the supertaxa are likewise preserved. For example,



                        filter_taxa(my_taxmap, taxon_names == 'Fungi', subtaxa = TRUE)
                    


would remove any taxa that are not named “Fungi” or are not a subtaxon of a taxon named “Fungi”. By default, steps are taken to ensure that the hierarchy remains intact when taxa are removed and that user-defined data are remapped to remaining taxa. When the reassign_taxa option is TRUE (the default), the subtaxa of removed taxa are reassigned to any supertaxa that were not removed, keeping the tree intact. When the reassign_obs option is TRUE (the default), any user-defined data assigned to removed taxa are reassigned to the closest supertaxa that passed the filter if such a taxon exists. This makes it easy to remove parts of the taxonomy without losing associated information. Finally, if the drop_obs option is TRUE (the default), any user-defined data assigned to removed taxa are also removed, allowing for subsetting of user-defined data based on taxon characteristics. The many combinations of these powerful options make filter_taxa a flexible tool and make it easier for new users to deal with the hierarchical nature of taxonomic data. For example, if the drop_obs option is TRUE (the default) and the reassign_obs option is FALSE, then any user-defined data assigned to taxa are removed even if a supertaxon is preserved. If the drop_obs option is FALSE, and the reassign_obs option is FALSE, then data associated with removed taxa is assigned a taxon ID placeholder of NA, but not removed. The function sample_n_taxa is a wrapper for filter_taxa that randomly samples some number of taxa. All of the options of filter_taxa can also be used for sample_n_taxa, in addition to options that influence the relative probability of each taxon being sampled.

Other dplyr analogs that help users manipulate their data include filter_obs, sample_n_obs, and mutate_obs.filter_obs is similar to running the dplyr function filter on a tabular, user-defined dataset, except that there are more values available to NSE and lists and vectors can also be subset. The drop_taxa option can be used to remove any taxa whose only observations have been removed during the filtering. The sample_n_obs function is a wrapper for filter_obs that randomly samples some number of observations. Like sample_n_taxa, there are options to weight the relative probability that each observation will be sampled. The mutate_obs function simply adds columns to tables of user-defined data.

***Mapping functions.*** There are also a few functions that create mappings between different parts of the data contained in taxmap or taxonomy objects. These are heavily used internally in the functions described already, but are also useful for the user. The subtaxa and supertaxa functions return the taxon IDs (or other values) associated with all subtaxa or supertaxa of each taxon. They return one value per taxon. The recursive option controls how many ranks below or above each taxon are traversed. For example, subtaxa(obj, recursive = 3) will return information for all subtaxa and their immediate subtaxa for each taxon. The recursive option also accepts a simple TRUE/FALSE, with TRUE indicating all subtaxa of subtaxa, etc., and FALSE only returning immediate subtaxa, but not their descendants. By default, subtaxa and supertaxa return taxon IDs, but the value option allows the user to choose what information to return for each taxon. For example, subtaxa(obj, value = "taxon_names") will return the names of taxa instead of their IDs. Any data available to NSE (i.e. in the result of all_names(obj)) can be returned in this way.

The functions roots, stems, branches, and leaves are a conceptual set of functions that return different subsets of a taxonomy. A “root” is any taxon that does not have a supertaxon. A “stem” is a root plus all subtaxa before the first split in the tree. A “branch” is any taxon that has only one subtaxon and one supertaxon. Stems and branches are useful to identify since they can be removed without losing information on the relative relationship among the remaining taxa. “Leaves” are taxa with no subtaxa. By default, these options return taxon IDs, but also have the value option like subtaxa and supertaxa, so they can return other information as well. For example, leaves(obj, value = "taxon_names") will return the names of taxa on the tips of the tree.

In the case of taxmap objects, the obs function returns information for observations associated with each taxon and its subtaxa. The observations could be rows in a table or elements in a list/vector that are named by taxon IDs. This is used to easily map between user-supplied information and taxa. For example, assuming a taxonomy with a single root, the value returned by obs for the root taxon will contain information for all observations, since they will all be assigned to a subtaxon of the root taxon. By default, row/element indices of observations will be returned, but the obs function also accepts the value option, so the contents of any column or other information associated with taxa can be returned as well.

***The parsers.*** Taxonomic data appear in many different forms depending on the source of the data, making parsing a challenge. There are two main sources of variation in how taxonomic data are typically stored: the type of information supplied (e.g. a taxon name vs. a taxon ID) and how it is encoded (e.g. in a table vs. as part of a string). In addition, there might be additional user-specific data associated with the taxa that need to be parsed. These data might be associated with each taxon in a classification (e.g the taxon ranks) or might be associated with each classification (e.g. a sequence ID). In many cases, both types are present. This complexity makes implementing a generic parser for all types of taxonomic data difficult, so parsers are typically only available for specific formats. The taxa package introduces a set of three parsing functions that can parse the vast majority of taxonomic data as well as any associated data and return a taxmap object.

The parse_tax_data function is used to parse taxonomic classifications stored as vectors in tables that have already been read into R. In the case of tables, the classification can be spread over multiple columns or in a single column with character separators (e.g. “ *Primates*; *Hominidae*; *Homo*; *sapiens*”) or a combination of the two. Other columns are preserved in the output and the rows are mapped to the taxon IDs (e.g. the ID assigned to “sapiens” in the above example). For both tables and vectors, additional lists, vectors or tables can be included and are assigned taxon IDs based on some shared attribute with the source of the taxonomic data (e.g. a shared element ID or the same order). This makes it possible to parse many data sets at once and have them all mapped to the same taxonomy in the resultant taxmap object. Data associated with each taxon in each classification can also be parsed and included in the output using regular expressions with capture groups identifying the information to be stored and a key corresponding to the capture groups that identifies what each piece of information is. For example, Hominidae_f_2;Homo_g_3;sapiens_s_4 would use the separator ";", the regular expression "(.+)_(.+)_(.+)", and the key c(my_taxon = "taxon_name", my_rank = "taxon_rank", my_id = "info"). The values of the key indicate what the information is (a taxon name and two arbitrary pieces of information) and the names of the key (e.g. “my_rank”) determine the names of columns in the output.

**Figure 2.**
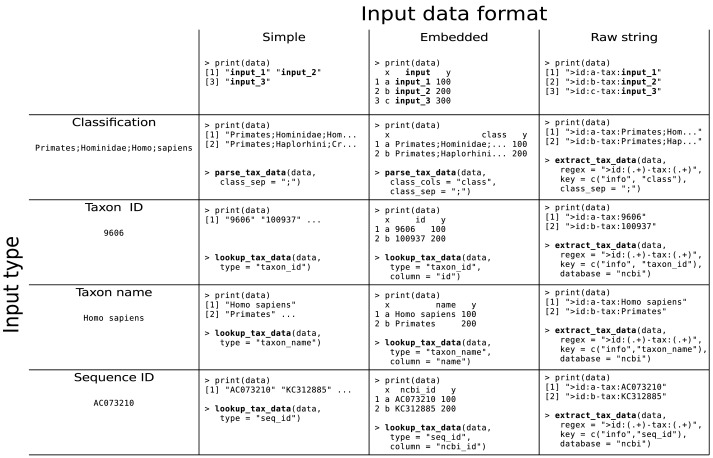
A table for determining how to parse different sources of taxonomic information using the taxa package. The rows correspond to the common sources of taxonomic information: full taxonomic classifications encoded in text, taxon IDs from a database, taxon names (a single rank), and NCBI sequence IDs. The columns correspond to the different formats the information can be encoded in: as a simple vector, as columns in a table, and as a piece of a complex string (e.g. a FASTA header). In the case of tables and complex strings, other information associated with the taxa can be preserved in the parsed result, as is done in the “use cases” example below. Any one cell in the table shows how to parse a given taxonomic information source in a given format using one of the three parsing functions: parse_tax_data, lookup_tax_data, extract_tax_data.

If only a taxon name (e.g. “Primates”) or a taxon ID for a reference database (e.g. the NCBI taxon ID for *Homo sapiens* is “180092”) is available in a table or vector, then the classification information must be queried from online databases and the function lookup_tax_data is used. lookup_tax_data has all the same functionality of parse_taxa_data in addition to being able to look up taxonomic classifications associated with taxon names, taxon IDs, and NCBI sequence IDs. If the data are embedded in a string (e.g. a FASTA header), then the function extract_tax_data is used instead. extract_tax_data has the functionality of parse_tax_data and lookup_tax_data, except that the information is extracted from raw strings using a regular expression and a corresponding key, the same way that data for each taxon in a classification is extracted by parse_tax_data. Together, these three parsing functions can handle every combination of data type and format presented in Figure 2 and many variations of those formats.

### Operation

Taxa is an R package hosted on CRAN, so only an R installation and internet connection are needed to install and use taxa. Once installed, most of the functionality of the package can be used without an internet connection. R can be installed on nearly any operating system, including most UNIX systems, MacOS, and Windows. The minimum system requirements of R and the taxa package are easily met by most personal computers. The amount of resources needed will depend on the size of data being used and the complexity of analyses being conducted. The package can be installed by entering install.packages("taxa") in an interactive R session. The development version can be installed from GitHub using the devtools package:



                        library
                        (devtools)

                        install_github(
                        "ropensci/taxa"
                        )
                    


**Figure 3.**
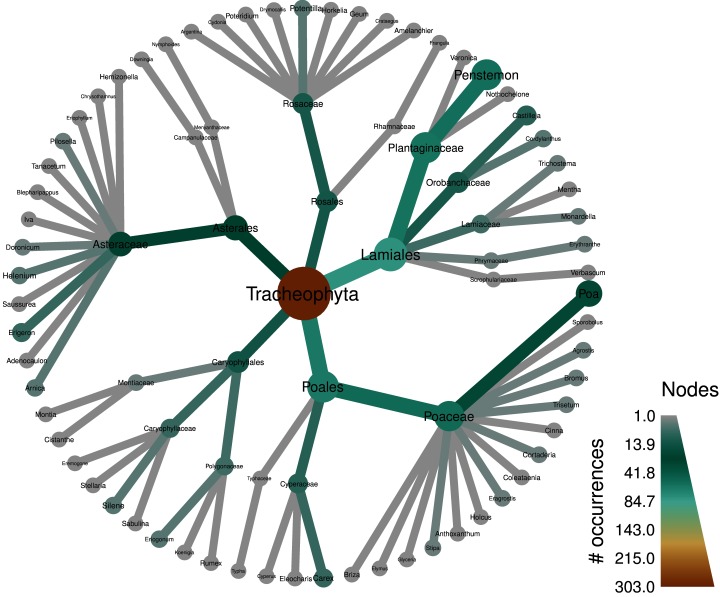
The result of the example analysis shown in the text. Records of plant species occurrences in Oregon are downloaded from the Global Biodiversity Information Facility (GBIF) using the rgbif package ( Chamberlain, 2017). Then a taxa parser is used to parse the table of GBIF data into a taxmap object. A series of filters are then applied. First, all occurrences that are not from preserved specimens as well any taxa that have no occurrences from preserved specimens are removed. Then, all taxa at the species level are removed, but their occurrences are reassigned to the genus level. All taxa without names are then removed. In the final two filters, only orders within Tracheophyta with greater than 10 subtaxa are preserved. The metacoder package is then used to create a heat tree (i.e. taxonomic tree) with color and size used to display the number of occurrences associated with each taxon at each level of the hierarchy.

For users, the typical operation of the software will involve parsing some kind of input data into a taxmap object using a method demonstrated in Figure 2. Alternatively, a dependent package, such as metacoder, might provide a parser that wraps one of the taxa parsers or otherwise returns a taxmap object. Once the data is in a taxmap object, the majority of a user’s interaction with the taxa package would typically involve filtering and manipulating the data using functions described in Table 1 and applying application-specific functions in other packages, such as metacoder ( Figure 3).

**Table 1.** Primary classes and functions found in taxa.

Function	Description
• taxon	A class that combines the classes containing the name, rank, and ID for a taxon.
• taxa	A simple list of taxon objects in an arbitrary order.
• hierarchy	A class that stores a list of nested taxa constituting a classification.
• hierarchies	A simple list of hierarchy objects in an arbitrary order.
• taxonomy	A class that stores a list of unique taxon objects and a tree structure.
• taxmap	A class that combines a taxonomy with user-defined, tables, lists, or vectors associated with taxa in the taxonomy. The taxonomic tree and the associated data can then be manipulated such that the two remain in sync.
• supertaxa • subtaxa	A "supertaxon" is a taxon of a coarser rank that encompasses the taxon of interest (e.g. *Homo* is a supertaxon of *Homo sapiens*). The "subtaxa" of a taxon are all those of a finer rank encompassed by that taxon. For example, *Homo sapiens* is a subtaxon of *Homo*. The supertaxa/subtaxa function returns the supertaxa/subtaxa of all or a subset of the taxa in a taxonomy object. By default, these functions return taxon IDs, but they can also return any data associated with taxa.
• roots • leaves • stems • branches	Roots are taxa that lack a supertaxon. Likewise, leaves are taxa that lack a subtaxon. Stems are those taxa from the roots to the first split in the tree. Branches are taxa with exactly one supertaxon and one subtaxon. In general, stems and branches can be filtered out without changing the relative relationship between the remaining taxa. By default, these functions return taxon IDs, but they can also return any data associated with taxa.
• obs	Returns the information about every observation from an user-defined data set for each taxon and their subtaxa. By default, indices of a list, vector, or table mapped to taxa are returned.
• filter_taxa • filter_obs	Subset taxa or associated data in taxmap objects based on arbitrary conditions. Hierarchical relationships among taxa and mappings between taxa and observations are taken into account.
• arrange_taxa • arrange_obs	Order taxon or observation data in taxmap objects.
• sample_n_taxa • sample_n_obs • sample_frac_taxa • sample_frac_obs	Randomly sample taxa or observation data in taxmap objects. Weights can be applied that take into account the taxonomic hierarchy and associated data. Hierarchical relationships among taxa and mappings between taxa and associated data are taken into account.

Since taxa provides highly flexible parsers, it is usually possible to convert data from other packages to taxa classes, enabling manipulation of that data by taxa functions or packages that build upon taxa, like metacoder. For example, using the general-use parsers provided by the taxa package, metacoder supplies specialized and easy to use parsers for the following formats: taxonomy files produced by mothur, biom files produced by QIIME and MEGAN, newick files, objects from the phyloseq package, phylo objects from the ape package, and fasta files from the Greengenes ( McDonald *et al.*, 2012), RDP ( Cole *et al.*, 2014), SILVA ( Yilmaz *et al.*, 2014), and UNITE databases ( Kõljalg *et al.*, 2013). We have not encountered any text-based file format containing taxonomic information that can be described using regular expressions that the taxa parsers cannot read. For classes from other packages that inherit list, vector, or data.frame, conversion is not needed to include that information in a taxmap object, since the manipulation functions such as filter_taxa will handle them correctly as is.

## Use case

Taxa is currently being used by metacoder and we are working on refactoring parts of taxize to work seamlessly with taxa as well. Both taxize and metacoder provide broadly useful functions such as querying databases with taxonomic information and plotting taxonomic information, respectively. We hope that having these two packages adopt the taxa framework will encourage developers of new packages to do so as well. Regardless, the flexible parsers implemented in taxa ( Figure 2) allow for data from nearly any source to be used. The example analysis below uses data from the package rgbif ( Chamberlain, 2017; Chamberlain & Boettiger, 2017), even though rgbif was not designed to work with taxa. This example shows a few of the benefits of using taxa. The function occ_data from the rgbif package returns a data.frame (i.e. table) of occurrence data for species from the Global Biodiversity Information Facility (GBIF) with one row per occurrence. The table has one column per taxonomic rank from kingdom to species.



                    # Look up plant occurrence data for Oregon

                    library
                    (rgbif)

                    occ 
                    <- 
                    rgbif::
                    occ_data
                    (stateProvince = 
                    "Oregon"
                    ,
                        
                     scientificName =
                    "Plantae"
                    )
                


This format returned by rgbif::occ_data is a variant on the format described in Figure 2, row 1, column 2, except that there is only one rank per column instead of all ranks being concatenated in the same column (the parser accepts any number of columns, each of which could contain multiple ranks delineated by a separator).



                    # Parse data with taxa

                    library
                    (taxa)

                    obj 
                    <- parse_tax_data
                    (occ
                    $
                    data, class_cols = 
                    c
                    (22:26, 28),           
 
                                         named_by_rank = TRUE)
                


In the taxmap object returned by parse_tax_data, the original table returned by occ_data is stored as obj$data$tax_data, but an extra column with taxon IDs for each row is prepended.



                    > 
                    print
                    (obj)

                    <Taxmap>

                    626 taxa: aab. Plantae ... ayc. NA

                    626 edges: NA->aab, aab->aac ... aml->ayc

                    1 
                    data 
                    sets:
  
                    tax_data:
    
                    # A tibble: 500 x 103
    
                    taxon_id name          key    decimalLatitude
      <chr>  <chr>         <int>  <dbl>
    1 amm    Racomitriu... 1.70e9 44.2
    2 amn    Orthotrich... 1.68e9 NA
    3 amo    Didymodon ... 1.67e9 45.7

                    # ... with 497 more rows, and 99 more

                    # <<< List of additional columns ommited >>>
                


The data are then passed through a series of filters piped together. The filter_obs command removes rows from the occurrence data table not corresponding to preserved specimens, as well as any corresponding taxa that no longer have occurrences due to this filtering. The multiple calls to filter_taxa that follow demonstrate some of the different parameterizations of this powerful function. By default, taxa that don’t pass the filter are simply removed and any occurrences assigned to them are reassigned to supertaxa that did pass the filter (e.g. occurrences for a deleted species would be assigned to the species’ genus). When the supertaxa option is set to TRUE, all the supertaxa of taxa that pass the filter will also be preserved. The subtaxa option works the same way. Finally, the filtered data are passed to a plotting function from the metacoder package that accepts the taxmap format. The plot is a taxonomic tree with color and size used to display the number of occurrences associated with each taxon ( Figure 3).



                    # Plot number of occurrences for each taxon

                    library
                    (metacoder)

                    obj %>%
  
                    filter_obs
                    (
                    "tax_data"
                    ,
              
                    basisOfRecord == 
                    "PRESERVED_SPECIMEN"
                    ,
              
                    drop_taxa = TRUE) %>%
  
                    filter_taxa
                    (taxon_ranks 
                    != 
                    "specificEpithet"
                    ) %>%
  
                    filter_taxa
                    (
                    ! is.na
                    (taxon_names)) %>%
  
                    filter_taxa
                    (taxon_names == 
                    "Tracheophyta",
               
                    subtaxa = TRUE) %>%
  
                    filter_taxa
                    (taxon_ranks == 
                    "order",
               
                    n_subtaxa > 10, subtaxa = TRUE,
               
                    supertaxa = TRUE) %>%
  
                    heat_tree
                    (node_label = taxon_names,
             
                    node_color = n_obs,
             
                    node_size = n_obs,
             
                    node_color_axis_label = 
                    "# occurrences"
                    )
                


Note the use of columns in the original input table like basisOfRecord being used as if they were independent variables. This is implemented by NSE as a convenience to users, but they could also have been included by typing the full path to the variable (e.g. obj$data$tax_data$basisOfRecord or occ$data$basisOfRecord). This is similar to the use of taxon_ranks and taxon_names, which are actually functions included in the class (e.g. obj$taxon_ranks()). The benefit of using NSE is that they are reevaluated each time their name is referenced. This means that the first time taxon_ranks is referenced in the example code it returns a different value than the second time it is referenced, because some taxa were filtered out. If obj$taxon_ranks() is used instead, it would fail on the second call because it would return information for taxa that have been filtered out already.

## Conclusions

While taxa is useful on its own, its full potential will be realized after being adopted by the community as a standard for interacting with taxonomic information in R. A robust standard for the commonplace problems of data parsing and manipulation will free developers to focus on specific novel functionality. The taxa package already serves as the foundation of another package called metacoder, which provides functions for plotting taxonomic information and parsing common file formats used in metagenomics research. Taxize, the primary package for querying taxonomic information from internet sources, is also being refactored to be compatible with taxa. We hope the broadly useful functionality of these two packages will jump start adoption of taxa as the standard for taxonomic data manipulation in R.

## Data and software availability

Install in R as install.packages("taxa")

Software available from: https://cran.r-project.org/web/packages/taxa/index.html

Source code available from: https://github.com/ropensci/taxa

Archived source code available from: https://doi.org/10.5281/zenodo.1183667 ( Foster *et al.*, 2017)

License: MIT
