# Supplementary material for: Taxa: An R package implementing data standards and methods for taxonomic data
Source: F1000Res. 2018 Sep 11;7:272. Originally published 2018 Mar 5. [Version 2] doi: 10.12688/f1000research.14013.2 (PMC5887078; doi:10.12688/f1000research.14013.2)
Supplement: Peer review history

# Peer review: Referee response for version 2

**DOI:** 10.5256/f1000research.17689.r38171

**Published:** 2018-11-12

**Authors:** Titus Brown

**Affiliations:** 1 Department of Population Health and Reproduction, University of California, Davis, Davis, CA, USA

This version addresses all of my concerns. Thank you!

I have read this submission. I believe that I have an appropriate level of expertise to confirm that it is of an acceptable scientific standard.

# Peer review: Referee response for version 2

**DOI:** 10.5256/f1000research.17689.r38170

**Published:** 2018-09-20

**Authors:** Holly M. Bik

**Affiliations:** 1 Department of Nematology, University of California, Riverside, Riverside, CA, USA

The authors have satisfactorily clarified all of my outstanding questions and added useful improvements to the text - additional explanations/discussions that makes this article informative for a broader audience were much appreciated.

I have read this submission. I believe that I have an appropriate level of expertise to confirm that it is of an acceptable scientific standard.

# Peer review: Referee response for version 2

**DOI:** 10.5256/f1000research.17689.r38173

**Published:** 2018-09-11

**Authors:** Ethan P. White, Kristina Riemer

**Affiliations:** 1 Department of Wildlife Ecology and Conservation and Informatics Institute, University of Florida, Gainesville, FL, USA; 2 Department of Wildlife Ecology and Conservation, University of Florida, Gainesville, FL, USA

This revision addresses all of our suggestions. Thanks!

We have read this submission. We believe that we have an appropriate level of expertise to confirm that it is of an acceptable scientific standard.

# Peer review: Referee response for version 1

**DOI:** 10.5256/f1000research.15231.r31496

**Published:** 2018-04-19

**Authors:** Holly M. Bik

**Affiliations:** 1 Department of Nematology, University of California, Riverside, Riverside, CA, USA

The authors present a framework named "taxa", designed to serve as a new standard package for interacting with taxonomy data in R. This package aims to address the ongoing difficulties in dealing with hierarchical taxonomy strings and numerical IDs in R, and I commend the authors on developing an exciting new framework that will simplify the manipulation and filtering of taxonomic data.

 Overall, I thought this was a well written manuscript that did a fairly comprehensive job at explaining the functions and classes within the taxa package. However, I have a few comments that would further clarify the package functionality and inputs, and help make this manuscript accessible to a more general audience of computational biologists and ecologists (e.g. with novice to intermediate knowledge of R).

- Currently, this manuscript is geared towards a technical audience who are experts in R programming and package development. I would incorporate some more generalized explanations of the taxa package and its purpose (e.g. that assume a novice level of knowledge in R). For example, the use case using GBIF data frames assumes that readers are familiar with the field of biodiversity informatics and the format/information content of GBIF species occurrence data.
- What is the ideal input file for the taxa package? A basic tab-delimited taxonomy mapping file (e.g. with Accession IDs and taxonomic hierarchies only), a metabarcoding OTU table (e.g. JSON formatted or tab-delimited from QIIME where taxonomy strings are embedded along with study-specific data), or a full database with accessions and associated taxonomic information such as SILVA or NCBI? This package seems like it offers powerful tools for parsing and manipulating taxonomic information but it is not entirely clear what end users could (or should) be using as input files.
- It would be useful to explain how the "taxa" package can be integrated and linked to the other ecological R packages. Specific explanations or use cases involving vegan or phyloseq would be useful here. The link to metacoder and taxize is much more clearly laid out, probably due to the fact that the authors also developed these packages.
- Related to the previous point, how would you use taxa as a standalone package? The use case examples presented here make it seem like the "taxa" package is much more useful when used in conjunction with metacoder or taxize. However, given the diverse functionality it seems like there are many other (very common) use cases for taxa that are not clearly presented here.
- How does "taxa" deal with (or allow manipulation / correction of) taxonomic hierarchies with non-homologous taxonomic levels. For example, a set of input hierarchies where level 4 represents "Order" level in Fungi but "Subclass" level in protists. This is a very common scenario for metabarcoding datasets - ideally you want to introduce gaps/placeholders for hierarchies that do not contain a certain level, so that users can automatically or manually standardize their taxonomic levels all rows in a dataset (e.g. making Level 7 correspond to "Family" level across all taxa).
- Does "taxa" (or related packages like taxize) contain any Taxonomic Name Resolution Service (TNRS) functionality? If not, is this planned for future releases?
- Page 5, paragraph 3: I found the description of the "reassign_taxa" option to be confusing. It was not clear to me what the purpose or result of  this reassignment function would be. Clarifying the wording and adding a real world example would be useful here.
- Table 1: The description of "arrange_taxa" and "arrange_obs" is fairly vague. Do these functions rearrange data within a file or object (e.g. sorting or filtering)? If so, what are the options for ordering data (e.g. by abundance, alphabetical sorting, etc.)

I have read this submission. I believe that I have an appropriate level of expertise to confirm that it is of an acceptable scientific standard, however I have significant reservations, as outlined above.

# Response

**Published:** 2018-08-31

**Authors:** Niklaus Grunwald

**Affiliations:** USDA ARS, USA

Thank very much for your detailed, constructive review that much improved this manuscript. We addressed all your comments as follows:

- “Currently, this manuscript is geared towards a technical audience who are experts in R programming and package development. I would incorporate some more generalized explanations of the taxa package and its purpose (e.g. that assume a novice level of knowledge in R). For example, the use case using GBIF data frames assumes that readers are familiar with the field of biodiversity informatics and the format/information content of GBIF species occurrence data.”

The paper was written mostly to package developers, but we agree that it would be valuable to make it more accessible. We added some more explanation of technical concepts, including the GBIF data.

- “What is the ideal input file for the taxa package? A basic tab-delimited taxonomy mapping file (e.g. with Accession IDs and taxonomic hierarchies only), a metabarcoding OTU table (e.g. JSON formatted or tab-delimited from QIIME where taxonomy strings are embedded along with study-specific data), or a full database with accessions and associated taxonomic information such as SILVA or NCBI? This package seems like it offers powerful tools for parsing and manipulating taxonomic information but it is not entirely clear what end users could (or should) be using as input files.”

The taxa package has no ideal input format and provides flexibility for many formats, but there are some formats that are much easier to parse than others. Tabular data is usually easy to read, as are delimited taxonomy strings (e.g. taxa separated by ;). The taxa parsers do not read files directly. Instead they parse data already in R, so the different transformations/subsets of data from the same file could be parsed differently. For this reason, taxa does not read JSON/BIOM files from QIIME. Instead, it provides highly abstracted parsers to handle most formats and provides the foundation for more specialized (and easier to use) parsers, like those found in metacoder, which wrap the taxa parsers. There is a parser for JSON/BIOM QIIME files (parse_qiime_biom) in metacoder that uses the taxa parsers internally. The other examples you listed are easily handled by the taxa parsers after reading the file into R using something like read.table.

- “It would be useful to explain how the "taxa" package can be integrated and linked to the other ecological R packages. Specific explanations or use cases involving vegan or phyloseq would be useful here. The link to metacoder and taxize is much more clearly laid out, probably due to the fact that the authors also developed these packages.”

`taxa` is primarily intended as a foundation for future packages rather than a way of interacting with existing packages, but it can use the data from other packages in some cases. For data structures from other packages that inherit lists, vectors, or data.frames, the taxa filtering functions should be able to manipulate them correctly as is. For other data structures with one or two dimensions with arbitrary row/column/item names, like vegan’s distance matrix or ape’s DNAbin objects, these can be included as is in the taxmap object’s `data` list, the same way standard data.frames/lists are, although the filtering functions do not currently support these and they will be ignored. We would like to add the ability to natively handle these classes in the future, so that, for example, a DNAbin could be included in a `taxmap` object and filtered using `filter_taxa` the same way a list can be now. This should not be too hard to implement, but we have not gotten to it yet. For complicated objects like phyloseq objects that hold many fields themselves, the best solution would be to convert them to `taxmap` objects, manipulate them with the taxa functions, and convert them back. The conversion should be lossless since the `taxmap` class should be able to store all the information in a phyloseq object. There is a function in metacoder called `parse_phyloseq` to convert a phyloseq object to a taxmap object and this uses the `taxa` parsers internally.

- “Related to the previous point, how would you use taxa as a standalone package? The use case examples presented here make it seem like the "taxa" package is much more useful when used in conjunction with metacoder or taxize. However, given the diverse functionality it seems like there are many other (very common) use cases for taxa that are not clearly presented here.”

Good point! Yes, `taxa` can be quite useful on its own. A few examples we can think of include:

- Looking up taxonomic classifications from sequence IDs, taxon IDs, and taxon names from a variety of databases (using taxize internally).
- Subsetting data to a specific taxon
- Removing ranks or specific taxa from classification strings
- Combining taxonomic data from multiple sources into the same taxonomy
- Getting lists of all the subtaxa/supertaxa for each taxon or other data associated with all of the subtaxa/supertaxa.

We added a few of these examples to the paper.

- “How does "taxa" deal with (or allow manipulation / correction of) taxonomic hierarchies with non-homologous taxonomic levels. For example, a set of input hierarchies where level 4 represents "Order" level in Fungi but "Subclass" level in protists. This is a very common scenario for metabarcoding datasets - ideally you want to introduce gaps/placeholders for hierarchies that do not contain a certain level, so that users can automatically or manually standardize their taxonomic levels all rows in a dataset (e.g. making Level 7 correspond to "Family" level across all taxa).”

In the `taxonomy` and `taxmap` classes, the taxonomy is stored as a tree structure, not a table, so rank information is not needed, although it is supported when present. The absence of a rank has no placeholder, it simply does not exist in the tree. If the user wanted to subset the tree to only taxa of a specific set of ranks, they could do something like `filter_taxa(obj, taxon_ranks %in% c(“family”, “genus”, “species”))` and the tree would remain intact, although there would be missing levels in the tree if some tips did not have a “family” supertaxon, for example. There is not currently a way to enforce that each rank exists at a fixed depth/level from the root of the tree, but we could add a function to add placeholder taxa to force that to the case (we added an issue to github at: https://github.com/ropensci/taxa/issues/169).

- “Does "taxa" (or related packages like taxize) contain any Taxonomic Name Resolution Service (TNRS) functionality? If not, is this planned for future releases?”

Yes, the parsing functions can optionally preprocess taxon names using the “Global Names Resolver” service via `taxize::gnr_resolve`. You just set the `type` option in `lookup_tax_data` or `extract_tax_data` to `”fuzzy_name”` instead of `”taxon_name”` to make this happen. This was a recent addition so it was not in the paper, but added it now.

- “Page 5, paragraph 3: I found the description of the "reassign_taxa" option to be confusing. It was not clear to me what the purpose or result of  this reassignment function would be. Clarifying the wording and adding a real world example would be useful here.”

Ok, we added some more explanation. The basic idea is that if you remove a taxon in the middle of the tree (say a family), it will assign any genera below that family to the order the family was in if reassign_taxa is set to TRUE (the default).

- “Table 1: The description of "arrange_taxa" and "arrange_obs" is fairly vague. Do these functions rearrange data within a file or object (e.g. sorting or filtering)? If so, what are the options for ordering data (e.g. by abundance, alphabetical sorting, etc.)”

"arrange_taxa" sorts the order of the taxa stored in `taxonomy` or `taxmap` objects. The order of taxa has little effect on most operations on these objects besides ordering the results of functions that return per-taxon information, like `supertaxa`. "arrange_obs" orders data stored in a `taxmap` objects (e.g. the row in an OTU table) based on some characteristics of that data. The options for ordering the data are therefore any piece of information associated with elements in that data set, such as the contents of columns in that data set (e.g. the name of OTUs, the counts of OTUs, etc). We added some more descriptions of this to the paper.

# Peer review: Referee response for version 1

**DOI:** 10.5256/f1000research.15231.r32711

**Published:** 2018-04-04

**Authors:** Peter Desmet, Damiano Oldoni

**Affiliations:** 1 Research Institute for Nature and Forest (INBO), Brussels, Belgium

The article is well written. Its structure is clear and the goals are well defined. Here below some minor issues.

**General Issue**

- What is the reason taxa functionalities are not implemented in taxize, which already seems a general purpose package to work with taxonomic information?

**Introduction**

- We found citation numbers for R to be very low. Does “its extensions” include all packages? Overall, it would be better to drop the sentence with citation numbers and keep it to R + easy development of packages, thus going fast to paragraph 2 which is more important to provide context for the taxa package
- “Database” is a very generic (technical) term. Would have expected “source”, or similar for source of taxonomic information, cf. **http://dublincore.org/documents/dcmi-terms/#elements-source**

**Methods**

 Implementation

- Figure 1: we found ourselves drawing examples of the classes presented in figure 1. Would maybe be useful to add those to figure? If it is not possible for graphic issues, maybe could be useful to add them in the text, more or less as done in vignette of the package.
- In “manipulation functions” : “Finally, if the drop_obs option is TRUE (the default), any user-defined data assigned to removed taxa are also removed, ...” With the reassign_taxa and reassign_obs discussed above, it wasn’t immediately clear how taxa can be removed. Maybe update to “... data assigned to removed taxa (those without supertaxa matching the criteria) are also removed ...”

**Use Cases**

- Use cases: one use case presented. Update title to “Use case”. The presented use case is very informative, no need to add more use cases
- Use case might have been stronger if taxonomic information from 2 sources was combined (e.g. GBIF and …)

Here below some minor issues about the package:

- Consider moving CONDUCT.md to .github directory, as that directory is already used for CONTRIBUTING.md
- Add proper MIT License in LICENSE file
- README.md is now a combination of https://github.com/ropensci/taxa/blob/master/vignettes/taxa-introduction.Rmd and https://github.com/ropensci/taxa/blob/master/README.Rmd. Would keep README shorter (based on README.Rmd), with links to vignettes instead.
- Consider adding a pkgdown website, with references for functions + the two vignettes. Site can be build in docs/ folder and hosted on GitHub pages, cf. https://inbo.github.io/wateRinfo/
- Add website to repo description in repo settings.
- Consider moving vignette figures to “vignettes/figures” subdirectory for clarity

We have read this submission. We believe that we have an appropriate level of expertise to confirm that it is of an acceptable scientific standard.

# Response

**Published:** 2018-08-31

**Authors:** Niklaus Grunwald

**Affiliations:** USDA ARS, USA

Thank very much for your detailed, constructive review that much improved this manuscript.  We addressed all your comments as follows:

- “What is the reason taxa functionalities are not implemented in taxize, which already seems a general purpose package to work with taxonomic information?”

The main focus of taxize is to download taxonomic information from online databases. Since there are many sources of taxonomic data, taxize is already a large and complicated package. The R community provides restrictions on the size of packages. Also, not all applications where a class system for taxonomic data is needed require the ability to download taxonomic information.

- “We found citation numbers for R to be very low. Does “its extensions” include all packages? Overall, it would be better to drop the sentence with citation numbers and keep it to R + easy development of packages, thus going fast to paragraph 2 which is more important to provide context for the taxa package”

It should include all packages. It does not seem low to us, considering that many people do not cite software, but we see your point. We removed it.

- ““Database” is a very generic (technical) term. Would have expected “source”, or similar for source of taxonomic information, cf. http://dublincore.org/documents/dcmi-terms/#elements-source”

We agree that “Source” would be a good term, although we hesitate to change the code at this point. We would have to rename the `taxon_database` to `taxon_source` (it is shorter, which is nice) and many option names that have some reference to “database”. Changing our references to “database” to “source” in the paper is easy enough, but then the different words used for the same thing in the paper and the code might confuse some people.

- “Figure 1: we found ourselves drawing examples of the classes presented in figure 1. Would maybe be useful to add those to figure? If it is not possible for graphic issues, maybe could be useful to add them in the text, more or less as done in vignette of the package.”

Good idea. We assume you are talking about the classes’ print methods. Some would fit well in Figure 1, but the `taxmap` and `taxonomy` print methods would be too big. We added some examples to the body of the paper.

- “In “manipulation functions” : “Finally, if the drop_obs option is TRUE (the default), any user-defined data assigned to removed taxa are also removed, ...” With the reassign_taxa and reassign_obs discussed above, it wasn’t immediately clear how taxa can be removed. Maybe update to “... data assigned to removed taxa (those without supertaxa matching the criteria) are also removed ...”

Yes, we see why that is confusing; thanks for the suggestion. Observations are only removed if they cannot be reassigned to something else. That could happen when “reassign_obs” is FALSE or there are no taxa left they could be reassigned to (as you say). We added some clarification about this.

- “Use cases: one use case presented. Update title to “Use case”. The presented use case is very informative, no need to add more use cases”

Good point, thanks!

- “Use case might have been stronger if taxonomic information from 2 sources was combined (e.g. GBIF and …)”

We like that idea, but we can't think of a way to do it that would keep the example simple. We could look up the taxonomic hierarchy from NCBI or ITIS using the species binomial, but it would be an odd thing to do when the full classification is already available, so it might make the example confusing.

- “Consider moving CONDUCT.md to .github directory, as that directory is already used for CONTRIBUTING.md”

Done, see: https://github.com/ropensci/taxa/issues/149

- “Add proper MIT License in LICENSE file”

To conform with CRAN guidelines we could not do this. See: https://github.com/ropensci/taxa/issues/150

- “README.md is now a combination of https://github.com/ropensci/taxa/blob/master/vignettes/taxa-introduction.Rmd and https://github.com/ropensci/taxa/blob/master/README.Rmd. Would keep README shorter (based on README.Rmd), with links to vignettes instead.” and “Consider adding a pkgdown website, with references for functions + the two vignettes. Site can be build in docs/ folder and hosted on GitHub pages, cf. https://inbo.github.io/wateRinfo/”

Good idea! We would like to add a website, but we will probably wait until we have one or two more vignettes done  (the second is still being worked on). Any additional vignettes will be added with links. We would be ok with reducing the readme once we have a website up with documentation up that we can link to.

https://github.com/ropensci/taxa/issues/151

- “Consider moving vignette figures to “vignettes/figures” subdirectory for clarity”

We will do so and have  pending issue on github: https://github.com/ropensci/taxa/issues/152

# Peer review: Referee response for version 1

**DOI:** 10.5256/f1000research.15231.r31493

**Published:** 2018-03-26

**Authors:** Titus Brown, Taylor Reiter

**Affiliations:** 1 Department of Population Health and Reproduction, University of California, Davis, Davis, CA, USA; 2 Food Science and Technology, University of California, Davis, CA, USA

The authors present the R package taxa, which provides a set of datatypes and functions for working with taxonomic data. The authors hope that they have contributed a strong base from which the taxonomic data ecosystem can build in R. The authors have also included a particularly useful set of parsers, and dplyr-like functionality within their package. The packages metacoder and rgbif are included as being compatible with taxa, and the authors mention that the popular package taxize is being refactored for compatibility. 

 Major points

- There is no discussion of the limitations of the software, or an specific discussion of incompatibility issues. If the authors have never encountered incompatibility issues, it would be helpful if they stated other packages or formats for which they have not encountered issues with. 
- The introduction does not provide a concrete discussion of the challenges that the package taxa addresses. 

Minor points

- In paragraph one, the authors note the ease with which one can develop an R package. I recommend adding "relative" somewhere in there.
- In paragraph two, it's not clear what is meant by "each package encodes this information differently." 
- In paragraph four, "Complexity ranges from simple," "simple" is perhaps not the right word
- In paragraph five, "However, using these classes allows for greater flexibility and rigor as the package develops," it is not clear what is meant by "the package."
- In paragraph six, "(e.g. Animalia, Chordata, Mammalia, Primates, Hominidae, Homo, sapiens)" and “Achlya” should be italicized. 
- I paragraph eight, "for the average user" should be removed. The clause, "that is easier for new users to understand than equivalent base R commands, which have accumulated some idiosyncrasies over the last 40 years" should also be rephrased to celebrate dplyr without cutting down base R. 
- In paragraph 10, "The many combinations of these powerful options make filter_taxa a flexible tool and make it easier for new users to deal with the hierarchical nature of taxonomic data," "make" should be "makes."
- In paragraph 11, the sentence "Other dplyr analogs that help users manipulate their data include filter_obs, sample_n_obs, and mutate_obs, filter_obs is similar to running the dplyr function filter on a tabular, user-defined dataset, except that there are more values available to NSE and lists and vectors can also be subset," is confusing.
- In paragraph 15, sentence 1, "for many users" should be removed.
- In paragraph 16, “Primates;Hominidae;Homo;sapiens,” “sapiens,” and "Primates" should be italicized
- In paragraph 17, "Together, these three parsing functions can handle every combination of data type and format (Figure 2)," every is a strong assertion.

We have read this submission. We believe that we have an appropriate level of expertise to confirm that it is of an acceptable scientific standard, however we have significant reservations, as outlined above.

# Response

**Published:** 2018-08-31

**Authors:** Niklaus Grunwald

**Affiliations:** USDA ARS, USA

Thank very much for your detailed, constructive review that much improved this manuscript.  We addressed all your comments as follows:

- “There is no discussion of the limitations of the software, or an specific discussion of incompatibility issues. If the authors have never encountered incompatibility issues, it would be helpful if they stated other packages or formats for which they have not encountered issues with.”

We think adding some information of limitations of the software is a good idea, but we are not sure what you had in mind exactly. In regards to limitations on data set size and speed, we have not explored this systematically yet, although we plan to identify parts of the code to port to C++ to increase speed where needed. We are also not sure what you mean specifically by “incompatibility issues”. It is certainly true that few packages are designed to work seamlessly with `taxa` currently, but `taxa` was designed with this in mind and the parsers can be used to import data from other formats not designed for use with `taxa`, as was done in the use case. We added some examples of packages we have used with `taxa` to demonstrate compatibility and will mention if we find any that are not compatible in some way.

 In regards to formats, in our own work and when helping others, we have used the taxa parsers with numerous ( > 20 maybe) different formats of taxonomic data and have never encountered a raw-text-based format that the current parsers cannot handle, but of course there might be some cases we have have not encountered/considered. We like the idea of adding a list of formats for which we have used taxa to read. We added this to the paper.

- “The introduction does not provide a concrete discussion of the challenges that the package taxa addresses.”

We do mention the lack of a  standard set of classes for packages to build on, which is the main challenge `taxa` is trying to address, and we added some more background on data parsing and manipulation, which are the other goals taxa` tries to address.

- “In paragraph one, the authors note the ease with which one can develop an R package. I recommend adding "relative" somewhere in there.”

Good point! We did that. We remember it did not appear easy when we started.

- “In paragraph two, it's not clear what is meant by "each package encodes this information differently.””

Ok, we added some examples.

- “In paragraph four, "Complexity ranges from simple," "simple" is perhaps not the right word”

Agreed. The low-level classes are quite simple currently, little more than containers for a few variables, but we removed the word “simple”, since it is not all that descriptive anyway.

- “In paragraph five, "However, using these classes allows for greater flexibility and rigor as the package develops," it is not clear what is meant by "the package."”

We meant `taxa`. We reworded that, thanks!

- “In paragraph six, "(e.g. Animalia, Chordata, Mammalia, Primates, Hominidae, Homo, sapiens)" and “Achlya” should be italicized.”

Agreed. Done.

- “I paragraph eight, "for the average user" should be removed. The clause, "that is easier for new users to understand than equivalent base R commands, which have accumulated some idiosyncrasies over the last 40 years" should also be rephrased to celebrate dplyr without cutting down base R.”

Agreed, we made those changes. We did not mean to berate base R, but rather point out a lack of consistency relative to dplyr, but we can see how people could get that impression.

- “In paragraph 10, "The many combinations of these powerful options make filter_taxa a flexible tool and make it easier for new users to deal with the hierarchical nature of taxonomic data," "make" should be "makes."”

“The many combinations” is plural, so we think it should be “make”.

- “In paragraph 11, the sentence "Other dplyr analogs that help users manipulate their data include filter_obs, sample_n_obs, and mutate_obs, filter_obs is similar to running the dplyr function filter on a tabular, user-defined dataset, except that there are more values available to NSE and lists and vectors can also be subset," is confusing.”

Thanks for catching that! That comma between “mutate_obs” and “filter_obs” was supposed to be a period, which we think makes it significantly less confusing.

- “In paragraph 15, sentence 1, "for many users" should be removed.”

Ok, we did that.

- “In paragraph 16, “Primates;Hominidae;Homo;sapiens,” “sapiens,” and "Primates" should be italicized”

Agreed.

- “In paragraph 17, "Together, these three parsing functions can handle every combination of data type and format (Figure 2)," every is a strong assertion.”

We meant every combination of data type and format covered in the preceding paragraphs and the figure, and we clarified that further.

# Peer review: Referee response for version 1

**DOI:** 10.5256/f1000research.15231.r31494

**Published:** 2018-03-26

**Authors:** Ethan P. White, Kristina Riemer

**Affiliations:** 1 Department of Wildlife Ecology and Conservation and Informatics Institute, University of Florida, Gainesville, FL, USA; 2 Department of Wildlife Ecology and Conservation, University of Florida, Gainesville, FL, USA

The software described in this paper provides useful tools for working with taxonomic data in R by providing a standard approach for storing and manipulating this hierarchically structured data. Taxonomic data is prevalent in many biological disciplines. As result, this package fills an important niche and has the potential to become widely used by other packages dealing with biological data.

 The software itself follows good development practices including modularization, documentation, version control, and automated testing. The package is available through CRAN – the main repository for R packages. Both the CRAN release and the development version of the package install smoothly. The use case examples given in the paper all run as expected on the development version, but they include functionality that is not present in the most recent release. This means that readers of the paper who have not installed the development version will encounter issues with the examples. We recommend a new minor version release so that the existing functionality is reflected in the latest release. Alternatively in-development functionality could be removed from the examples in the paper.

 The paper does a nice job of motivating the need for the package and the use case section nicely demonstrates some of the core functionality. However, there are improvements that could be made to help the paper communicate with a broader audience. Specifically we recommend changes to the Introduction and Methods sections.

 In the Introduction we suggest either expanding the second paragraph or adding a new paragraph to describe the other kinds of datasets that this will be helpful for. There are many large and small ecological and evolutionary datasets beyond high-throughput sequencing that involve lots of taxonomic data (e.g., museum records, citizen science projects, compilations of literature data) and broadening the context will help more readers understand why this package might be useful to them. We also suggest adding an additional paragraph, following the third paragraph, that describes typical taxonomic data, including an example, and that mentions the specific challenges of this kind of hierarchical data. This will help readers less familiar with these issues understand the value of the software and help set up the technical details in the last paragraph of the Introduction. To make room for these additions we suggest removing the first paragraph, which currently states that R is “becoming the leading tool for scientific data analysis in academic research.” This specific interpretation isn’t justified by the associated citation and it is broadly understood that R is an important language so a paragraph explaining this isn’t really necessary.

 In the Methods we suggest moving the parsing section to the beginning, and using the examples from that section throughout the descriptions of classes. This will help ground the descriptions of the classes and how they are related, which currently reads as somewhat abstract. The current second paragraph (“The hierarchy and taxonomy class”) would benefit from having the hierarchy class defined more and the differences between the hierarchy and taxonomy classes clarified. For example, it is stated later that the hierarchy class is simpler and the taxonomy class is more hierarchical; it would be helpful to include this information earlier. Moving the last two sentences of this paragraph to the beginning might address this issue. The taxon IDs information could be its own paragraph, starting with “Using automatically generated taxon IDs”. The examples in that section are really helpful. In the beginning of the third paragraph of the methods (“The taxmap class”), it would be helpful to emphasize that this class combines the rest of the original data (including an example of original data, e.g., mass) back with the taxon class. Finally, a figure of an example taxonomic hierarchy that illustrates the operation of the filtering, mapping, and roots/stems/branches/leaves functions would be useful.

**Minor suggestions**

- Define the following phrases to broaden communication
  - “character vectors”, first paragraph of methods
  - “custom print method”, first paragraph of methods
  - “non-standard evaluation”, third paragraph of methods
  - “parsing”, paragraph 11 of methods
- Cite Wickham & Francois (2015) for the dplyr philosophy in the fourth Methods paragraph
- Consider color coding the boxes in Fig. 1 to match the three classes paragraphs
- Define R6 and S3 in the Fig. 1 legend

We have read this submission. We believe that we have an appropriate level of expertise to confirm that it is of an acceptable scientific standard.

# Response

**Published:** 2018-08-31

**Authors:** Niklaus Grunwald

**Affiliations:** USDA ARS, USA

Thank very much for your detailed, constructive review that much improved this manuscript.  We addressed all your comments as follows:

- ‘The use case examples given in the paper all run as expected on the development version, but they include functionality that is not present in the most recent release. This means that readers of the paper who have not installed the development version will encounter issues with the examples. We recommend a new minor version release so that the existing functionality is reflected in the latest release.”

Thanks for catching this! We have released a version on CRAN with this functionality.

- “In the Introduction we suggest either expanding the second paragraph or adding a new paragraph to describe the other kinds of datasets that this will be helpful for. There are many large and small ecological and evolutionary datasets beyond high-throughput sequencing that involve lots of taxonomic data (e.g., museum records, citizen science projects, compilations of literature data) and broadening the context will help more readers understand why this package might be useful to them”

Good idea! We added a section on this.

- “We also suggest adding an additional paragraph, following the third paragraph, that describes typical taxonomic data, including an example, and that mentions the specific challenges of this kind of hierarchical data. This will help readers less familiar with these issues understand the value of the software and help set up the technical details in the last paragraph of the Introduction”

Ok, good idea, we added some examples of diverse formats that we have used.

- “To make room for these additions we suggest removing the first paragraph, which currently states that R is “becoming the leading tool for scientific data analysis in academic research.” This specific interpretation isn’t justified by the associated citation and it is broadly understood that R is an important language so a paragraph explaining this isn’t really necessary.”

Yes, we had a similar comment from another reviewer, so we removed part of this.

- “In the Methods we suggest moving the parsing section to the beginning, and using the examples from that section throughout the descriptions of classes. This will help ground the descriptions of the classes and how they are related, which currently reads as somewhat abstract.”

The parsers only returns `taxmap` objects so far, and `taxmap` is built upon the previous classes, so that is why we ordered it that way. However, we agree that it is not immediately clear what the importance of the first classes described are.

- “The current second paragraph (“The hierarchy and taxonomy class”) would benefit from having the hierarchy class defined more and the differences between the hierarchy and taxonomy classes clarified. “

Agreed, we will clarify this.

- “In the beginning of the third paragraph of the methods (“The taxmap class”), it would be helpful to emphasize that this class combines the rest of the original data (including an example of original data, e.g., mass) back with the taxon class. “

Good point. This is a key aspect of the class.

- “Finally, a figure of an example taxonomic hierarchy that illustrates the operation of the filtering, mapping, and roots/stems/branches/leaves functions would be useful.”

We like this idea. This is something we have considered in the past. We will add this to one of our vignettes in the future:

 https://github.com/ropensci/taxa/issues/170 

- “Define the following phrases to broaden communication
- “character vectors”, first paragraph of methods
- “custom print method”, first paragraph of methods
- “non-standard evaluation”, third paragraph of methods
- “parsing”, paragraph 11 of methods

Agreed, we made those changes.

- “Cite Wickham & Francois (2015) for the dplyr philosophy in the fourth Methods paragraph”

We cited it in the introduction.

- “Consider color coding the boxes in Fig. 1 to match the three classes paragraphs”

We are not sure what you mean here.

- “Define R6 and S3 in the Fig. 1 legend”

Agreed.
